# Utility of an alternative bicycle commute route of lower proximity to motorised traffic in decreasing exposure to ultra-fine particles, respiratory symptoms and airway inflammation – a structured exposure experiment

**DOI:** 10.1186/1476-069X-12-29

**Published:** 2013-04-08

**Authors:** Tom Cole-Hunter, Rohan Jayaratne, Ian Stewart, Matthew Hadaway, Lidia Morawska, Colin Solomon

**Affiliations:** 1 Institute of Health and Biomedical Innovation, Queensland University of Technology, 60 Musk Avenue, Brisbane, QLD 4059, Australia; 2 International Laboratory for Air Quality and Health, Queensland University of Technology, 2 George Street, Brisbane, QLD 4001, Australia; 3 School of Life Sciences, Queensland University of Technology, 2 George Street, Brisbane, QLD 4001, Australia; 4 School of Health and Sport Sciences, University of the Sunshine Coast, Sippy Downs Drive, Sippy Downs, QLD 4556, Australia

**Keywords:** Air pollution, Bicycle commuting, Ultrafine particle, Respiratory symptom, Lung function, Inflammatory cell, Risk management

## Abstract

**Background:**

Bicycle commuting in an urban environment of high air pollution is known to be a potential health risk, especially for susceptible individuals. While risk management strategies aimed to reduce exposure to motorised traffic emissions have been suggested, only limited studies have assessed the utility of such strategies in real-world circumstances.

**Objectives:**

The potential to lower exposure to ultrafine particles (UFP; < 0.1 μm) during bicycle commuting by reducing proximity to motorised traffic was investigated with real-time air pollution and intermittent acute inflammatory measurements in healthy individuals using their typical higher proximity, and an alternative lower proximity, bicycle commute route.

**Methods:**

Thirty-five healthy adults (mean ± SD: age = 39 ± 11 yr; 29% female) completed two return trips, one each in the condition of their typical route (HIGH) and a pre-determined alternative route of lower proximity to motorised traffic (LOW); proximity being determined by the proportion of on-road cycle paths. Particle number concentration (PNC) and diameter (PD) were monitored in-commute in real-time. Acute inflammatory indices of respiratory symptoms (as a scalar of frequency from very low to very high / 1 to 5), lung function and spontaneous sputum (for inflammatory cell analyses) were collected immediately pre-commute, and immediately and three hours post-commute.

**Results:**

In the condition of LOW, compared to in the condition of HIGH, there was a significant decrease in mean PNC (1.91 x e^4^ ± 0.93 × e^4^ ppcc vs. 2.95 × e^4^ ± 1.50 × e^4^ ppcc; p ≤ 0.001), and the mean frequency of in-commute offensive odour detection (2.1 vs. 2.8; p = 0.019), dust and soot observation (1.7 vs. 2.3; p = 0.038) and nasopharyngeal irritation (1.5 vs. 1.9; p = 0.007). There were no significant differences between LOW and HIGH in the commute distance and duration (12.8 ± 7.1 vs. 12.0 ± 6.9 km and 44 ± 17 vs. 42 ± 17 min, respectively), or other indices of acute airway inflammation.

**Conclusions:**

Exposure to PNC and offensive odour, and nasopharyngeal irritation, can be significantly lowered when utilising a route of lower proximity to motorised traffic whilst bicycle commuting, without significantly affecting commute distance or duration. This may bring health benefits for both healthy and susceptible individuals.

## Introduction

The health benefits of physical activity associated with active transport are well-established [1-3], but the negative health effects of elevated air pollution exposure are also known [4-6]. Subsequently, there is evidence for cause-effect mechanisms and methods to reduce the degree of air pollution exposure whilst actively commuting [7-11]. Risk management strategies for reducing air pollution exposure whilst actively commuting can include reducing proximity to motorised traffic by avoiding main roads at peak traffic times [12]. The majority of projects on this topic have utilised micro-environments of designated off-road and on-road bicycle paths, and have determined that the former generally facilitates a significantly lower potential for exposure to air pollution, mainly from motorised traffic emissions such as ultrafine particles (UFP) [13-17]. Health indices, including acute respiratory symptoms, impaired lung function and inflammation-associated cell distribution have been used to investigate the physiological response to components of air pollution including particle number concentrations (PNC; which is dominated by UFP) [18-20].

Additionally, questionnaires that assess the influence of air pollution exposure on the airways have been used previously [21-24], using specific symptoms attributable to acute air pollution exposure as recommended by the American Thoracic Society [25]. For example, airway narrowing due to inflammation and excessive mucous secretion (as an immune response to airway irritation by pollutants) can induce coughing and chest tightness or wheezing, as well as reduce lung function indicated by lowered peak expiratory flow rates [26]. Further, an increase in the number of leukocytes, and specifically neutrophils, found in the airways and systemic circulation can indicate an inflammatory response to exposure from air pollutants such as ultrafine particles [27,28]. However, the utility by bicycle commuters of an informed alteration to their own typical route to avoid major motorised traffic corridors, and consequently reduce exposure to elevated PNC, and thereby decrease any associated negative health effects, is yet to be investigated.

Bicycle commuters may not have a route which allows complete use of off-road bicycle paths. Therefore, it is not expected to be practical for a bicycle commuter to completely alter their commute route and completely avoid exposure to motorised traffic emissions, particularly those due to factors such as road crossings that dissect off-road paths. However, it is possible, and may be practical, to decrease exposure to UFP by selecting a route which has reduced proximity to motorised traffic. Therefore, for this experiment it was hypothesised that: 1) a route alteration designed to lower proximity to motorised traffic during bicycle commuting will significantly reduce exposure to combustion emissions [represented by the dominant ultrafine particle (UFP; < 0.1 μm) number concentration (PNC)], compared to a high proximity route; 2) health outcomes, including incidence and severity of acute respiratory symptoms, peak flow rate, and cell distribution in sputum, will be improved with the use of a route of lower proximity to motorised traffic, compared to a route of high proximity; 3) the difference in the estimated inhaled PNC between the two routes will be attributable to the difference in ambient UFP, rather than any difference in physical effort (indicated by heart and ventilation rate).

## Methods

### Project design

This project was designed to determine whether the use of an alternative bicycle commute route designed to lower proximity to motorised traffic (and therefore lower exposure to associated emissions) is practical as an exposure risk management strategy. Thirty-five healthy adults were recruited to perform their typical workday commute along both their typical route (deemed as of high proximity to motorised traffic; termed HIGH) and an altered route (designed to be of lower proximity to motorised traffic; termed LOW). The participants and their bicycles were instrumented to measure real-time exposure variables of geolocation, heart rate, and particle number concentration and diameter while in-commute. Participants performed a selection of physiological inflammatory response tests immediately pre-commute, immediately post-commute and three hours post-commute to relate these outcomes to in-commute exposure variables. This project was approved by the Queensland University of Technology Human Research Ethics Committee. Prior to participation in this project, written informed consent was provided by participants.

### Participants

The participants of this project were healthy adults (n = 35; 29% female. Mean ± SE: age = 39 ± 11 yr, PFR = 558 ± 105 L·min) with no history of cardiopulmonary disease and no recent history of smoking (cessation > twenty-four months prior) or respiratory infection (symptoms > two weeks prior). Participants were required to be frequent bicycle commuters of the Brisbane inner-city region [defined as completing two or more return trips in a five day period to a destination within a 1 km radius of the Brisbane Central Business District (CBD)] and have a typical commute route of high proximity to motorised traffic. Recruitment was conducted from participants who provided consent as part of a previous study [22], and eligible respondents to a regional media release. Participants were requested to avoid any air pollution sources where possible, such as second-hand smoke and traffic congestion during the pre-commute and three hour post-commute monitoring period. This request may have affected a participant’s typical activity and exposure, however it was included to concentrate any acute inflammatory response to the in-commute exposure.

### Project locality

Brisbane is the state capital of Queensland, and the third largest city in Australia. The Brisbane CBD is located at 27º3^′^ South, 153º9^′^ East, approximately 20 km inland from the Pacific Ocean. Brisbane is located in a low-lying floodplain, with several large hills of up to 300 metres in height within the area, bordered to the west by a coastal mountain range. A large river runs through the city area. The regional climate is sub-tropical, being cool and dry in winter (between June to August), and humid and wet in summer (between December to February) [29]. The city of Brisbane has a population of approximately two million people, which has been increasing annually by approximately two percent for the last two decades [30]. Motorised traffic volume, along with population growth, is rapidly increasing, particularly due to outer-city residential development [30]. The number of motor vehicles registered within Brisbane in 2011 was approximately one million, however the greater region of South-East Queensland includes a total of nearly three million [31]. Industrial air pollution sources include a major airport, seaport, and oil refineries (all approximately 15 km north-east of the CBD), a coal power station (approximately 30 km south-west of the CBD), and various manufacturing companies in the outer suburbs.

### Routes of high and low proximity to motorised traffic

The participants typical commute route, which had been selected due to it being more than two-thirds on-road cycle path, was used as the high proximity to motorized traffic (HIGH) route. Participants, in consultation with the primary investigator, determined the altered route of lower proximity to motorised traffic (LOW) based on their typical bicycle commute route. Each participant rode a return trip [inbound (morning) and outbound (evening)] of each of HIGH and LOW on consecutive days. An equal number of participants performed HIGH or LOW first, to counter-balance and negate any influence of the order of the route condition. Therefore, a total of 140 trips were performed as a result of 35 participants each completing an inbound-HIGH, outbound-HIGH, inbound-LOW and outbound-LOW trip. Following the completion of these two return trips, participants were asked which route (HIGH or LOW) they preferred and to rate the importance of specific components (with examples) such as fitness (e.g. longer training session), health (e.g. to avoid air pollution), safety (e.g. greater riding space / visibility), social (e.g. group commute / drink stop) and time (e.g. more convenient / quickest route) each on a five-grade scale (1 = Very low, 2 = Low, 3 = Moderate, 4 = High, 5 = Very high).

### In-commute particle concentration and diameter

To measure and record real-time PNC and diameter (PD) both in-commute and three hours post-commute, an Aerasense NanoTracer (Philips, The Netherlands) with a 16-second logging frequency was carried by each participant. The sampling tube of the NanoTracer was attached to the participant in their immediate breathing zone, for example on their shirt collar or upper backpack strap. The NanoTracer is a compact and portable device capable of measuring PNC (0 – e^6^ particles per cubic centimeter (ppcc)) and PD (0.01 – 0.3 μm) in real-time via diffusion charging [32]. The NanoTracer does not experience tilt errors that may be produced with fluid-reliant instruments (such as a condensation particle counter) during vigorous use (such as for active transport monitoring); therefore, this device has potential for application in the field, which has been shown through previous research [33]. In-commute PNC and PD means, medians and range were calculated with NanoReporter software (Philips, The Netherlands). Individual device correction factors of 0.75, 0.99 and 1.30 for each of the three NanoTracers used were determined by laboratory testing at the International Laboratory for Air Quality and Health (ILAQH) using a condensation particle counter (CPC 3787, TSI) in atmospheric air (at a university campus location 200 metres from a main highway with four traffic lanes in each direction) for 4 hours. PNC and PD means below 100 ppcc and 0.01 μm, respectively, or that changed by more than a factor of 10 at subsequent readings, are considered to be invalid and thus were removed from initial particle measurement data prior to analyses [10,34]. The NanoTracer is factory-calibrated, so any attempt of self-calibration was not possible (due to risk of warranty nullification) and was beyond the scope of this study.

### In-commute heart rate

Heart rate (F_H_) was monitored, both in-commute and for three hours post-commute, using a telemetry unit (Polar Electro, Finland) logging at five second intervals. In-commute F_H_ was compared between HIGH and LOW to determine if there was a difference in physical effort when performing the two routes and therefore indicate if an inhaled particle count is affected most by the PNC in the air or the ventilation rate for the two routes. As an individual’s F_H_ and ventilation rate are associated [35], a higher mean trip F_H_ would produce a higher mean trip ventilation rate and therefore a higher total number of inhaled particles at any PNC.

### Climate

The Australian Bureau of Meteorology Climate Database [29] was accessed for hourly regional measures of temperature, humidity, wind direction and speed, air pressure, and precipitation. Meteorological data was collated and analysed to explain any particle measurement differences between commute monitoring days due to different atmospheric conditions.

### Physiological inflammatory responses

The participants performed three self-administered tests (as symptom reporting, peak flow metering and sputum collecting) to assess the manifestation of an acute biological inflammatory response attributable to air pollution exposure. A verbal and written explanation of instructions for each test was provided to participants at an induction session. Symptoms and peak flow rates were measured immediately pre-commute and post-commute, and three hours post-commute either at the participant’s home or workplace. Sputum samples were collected immediately pre-commute and three hours post-commute only. See Additional files 1 and 2 for written instructions and symptom questionnaire provided to participants.

#### Symptom questionnaire

At the induction, participants were supplied with a questionnaire (produced using information from the American Thoracic Society [25]) to report the incidence and severity of specific signs and symptoms including offensive odour detection (and dust or soot observation), eye, nose and throat irritation, coughing and/or phlegm production, chest tightness and/or wheezing, on a five-grade scale (1 = Very Low, 2 = Low, 3 = Moderate, 4 = High, and 5 = Very High). The same questions were used for each time period of immediately pre-commute, immediately post-commute, and three hours post-commute to attribute symptoms to air pollution data of each monitored commute trip.

#### Peak expiratory flow rates

At the induction, participants were supplied with a peak flow meter (MicroPeak, CareFusion, UK) and disposable one-way mouthpieces. Participants were instructed to perform and record three peak flow maneuvers (to obtain a mean value) at the end of each time period of immediately pre-commute, immediately post-commute, and three hours post-commute to relate peak flow changes to air pollution data of each monitored trip. The standard deviations of peak flow rates were examined to evaluate reproducibility of the forced expiratory manoeuvre.

#### Sputum sampling and cell counts

At the induction, participants were supplied with plastic (Falcon) tubes (15 mL) containing 2 mL of an RNA collection solution (RNAlater), the relevant RNAlater material safety data sheet, and instructions for spontaneous sputum production. Approximately 2 mL of sputum was collected in the RNAlater and immediately refrigerated at approximately 4°C by participants, and then frozen at -20°C within 24 hours by investigators for later analysis.

For total cell counts, the sputum samples were removed from the -20°C freezer and thawed, then centrifuged for 15 min at 500 x g at 25°C. The supernatant was removed and the cell pellet maintained, then 2 mL of PBS was added to suspend the cells and the sample was briefly vortexed. The cell suspension was aliquoted (2 × 10 μL) in to the haemocytometer slide wells, and then viewed at x40 magnification (Olympus light microscope). Satisfactory total cell counts were completed by one blinded counter. The proportion of squamous epithelial cells (SECs) was determined to indicate validity via saliva contamination (by ≤ 400 SECs per 100 leukocytes) of each sputum sample.

For differential cell counts, a 200 μL aliquot of cell suspension was cytospun (5 min at 100 x g), fixed with methanol, stained (Rapid Diff) and mounted (Permount) with a cover-slip, and then viewed at x100 magnification (Olympus light microscope). Satisfactory differential cell counts (from 2 slides with >50% viability and <75% SECs, 100 non-SECs per slide) were completed by one blinded counter.

Cell count reference values of eosinophils, lymphocytes, macrophages and neutrophils in induced sputum of healthy adults were consulted to inform validation of sample quality [36].

### Statistical data analysis

Due to the expected variation of typical commute characteristics (including commute time, distance and duration) within the participant group, the in-commute trip variable means (as well as medians for PNC and PD) of the four different individual data sets (i.e. both inbound and outbound trips of HIGH and LOW) of a single participant were initially compared. Subsequently, means (or medians for PNC and PD) of both inbound and outbound trips of HIGH and LOW were compared to determine the utility of a bicycle commute route alteration to lower exposure to motorised traffic-emitted ultrafine particles. All analyses were performed with predictive analytics software (PASW v18.0; IBM, New York).

Estimated marginal means of personal factors and factors of exposure, along with descriptive values, were produced. Pearson bivariate correlations were performed for particle measurements (PNC, PD) with independent variables of meteorology, symptoms and age. Pearsons bivariate correlations were also performed between CBD proximity (indicative of general proximity to motorised traffic) and HIGH and LOW data for PNC, PD and heart rate. Multivariate repeated measure ANOVA was performed with the mean and median of the dependent variables of PNC and PD for both inbound and outbound conditions of HIGH and LOW to determine intra-individual variability. One-way ANOVA, and Tukey Post-Hoc tests, where applicable, were performed with PNC, PD (as independent variables), gender and the dependent variable symptom reporting, each at the three different time-points. Mixed Effects Models analysis was performed with PNC, PD and participant symptom reporting, lung function and cell counts to determine the effect of particles on the physiological inflammatory response between inbound and outbound in HIGH and LOW. Mixed Effects Model analysis was performed to determine the difference between the three time-points within each commute condition (i.e. immediately pre-commute, immediately post-commute and three-hours post-commute) in relation to in-commute PNC and PD. Statistical significance was accepted at P < 0.05.

## Results

### Bicycle commute characteristics

Due to the regional location of the bicycle paths, it was not practically possible to produce exactly the same proportion of off-road paths for each participant; therefore, as expected, there was a range in the distribution of path type within HIGH and LOW. For example, popular south and west LOW routes were adjacent to, but physically-separated from, two different major motorised traffic corridors and therefore had lower proportions of off-road paths. Conversely, popular north and east LOW routes ran adjacent to parklands and a major river, respectively, facilitating higher proportions of off-road paths.

### In-commute particle measurements

The mean commute PNC for LOW was significantly lower than HIGH [1.91 × e^4^ ± 0.93 × e^4^ vs. 2.95 × e^4^ ± 1.50 × e^4^ ppcc; F-statistic (degrees of freedom) and p-value: F(1,35) = 21.079 and p ≤ 0.001], and the mean commute PNC within HIGH was significantly higher for inbound compared to outbound trips [F(1,35) = 8.441; p = 0.007]. See Table 1. The median commute PNC for LOW was significantly lower than HIGH [1.36 × e^4^ ± 0.73 × e^4^ vs. 1.99 × e^4^ ± 1.05 × e^4^ ppcc; F(1,35) = 14.025; p = 0.001], however there was no significant difference between LOW or HIGH inbound and outbound trips (p > 0.10). See Table 1.

**Table 1:** Commute variables for routes of high (HIGH) and low (LOW) proximity to motorised traffic; direction

**Condition**	**HIGH**		**LOW**	
	**Inbound**	**Outbound**	**Inbound**	**Outbound**
Time (24:00)	8:20 ± 0:22	16:39 ± 0:23	8:04 ± 0:22	16:33 ± 0:23
Distance (km)	12.3 ± 6.9	11.7 ± 6.9	12.9 ± 7.2	12.6 ± 7.0
Duration (min)	42 ± 18	41 ± 15	45 ± 17	43 ± 16
Speed (km·hr^-1^)	17.3 ± 4.3	16.7 ± 4.8	17.1 ± 4.6	17.1 ± 4.7
Heart Rate (bpm)	137 ± 11	135 ± 11	134 ± 9	131 ± 9
Temperature (ºC)	17.9 ± 3.5	21.1 ± 3.0^##^	17.7 ± 3.5	21.5 ± 3.2^##^
Humidity (%)	61 ± 14	48 ± 19^##^	62 ± 13	49 ± 19^##^
Wind Speed (km·hr^-1^)	5.8 ± 3.2	9.5 ± 4.8	7.1 ± 3.2	8.5 ± 4.4
Air Pressure (hPa)	1019 ± 6	1016 ± 5	1019 ± 6	1016 ± 5
PNC Mean ( x e^4^; ppcc)	3.30 ± 1.57	2.60 ± 1.35^##^	1.99 ± 1.02^**^	1.84 ± 0.84^**^
PNC Median ( x e^4^; ppcc)	2.20 ± 1.02	1.77 ± 1.08	1.34 ± 0.79^**^	1.38 ± 0.67^**^
PD Median (nm)	49 ± 10	47 ± 8	52 ± 11	50 ± 11

Values are means (or median where indicated) ± standard deviation. Significance [from multivariate repeated measure ANOVA]: ^*^p < 0.05, ^**^p < 0.01 compared to HIGH; ^#^p < 0.05, ^##^p < 0.01 compared to Inbound. PNC (ppcc) = particle number concentration (particles per cubic centimetre), PD = particle diameter.

The median commute PD was not significantly different between HIGH and LOW, or between HIGH or LOW inbound and outbound trips (p > 0.08). See Table 1. Mean PNC and PD were negatively-correlated (r = -0.645; p = 0.048).

### Commute distance, duration, speed and heart rate

The mean commute distance and duration, and therefore commute speed, were not significantly different between HIGH and LOW [12.0 ± 6.9 vs. 12.8 ± 7.1 km (p > 0.10) and 42 ± 17 vs. 44 ± 17 min (p > 0.10), 17.0 ± 4.6 vs. 17.1 ± 4.7 km/hr (p > 0.10)], or between HIGH or LOW inbound and outbound trips. See Table 1. The mean commute heart rate were not significantly different between HIGH and LOW, or between HIGH or LOW inbound and outbound trips, (p > 0.10). See Table 1.

### Climate

All meteorological variables were not significant different between HIGH and LOW (Data not presented, p > 0.10). Due to natural diurnal variation, the mean inbound (morning) commute temperature was significantly lower [F(1,35) = 47.085; p ≤ 0.001] and the humidity significantly higher [F(1,35) = 54.114; p ≤ 0.001], compared to the outbound (afternoon) trip. See Table 1.

Mean commute temperature was negatively-correlated with PNC (r = -0.83; p = 0.005) and positively-correlated with PD (r = 0.79; p = 0.014). Mean commute humidity was not significantly correlated with mean PNC or PD (both with r < 0.40; p > 0.10). Regional wind direction was not correlated to particle measurements (r < 0.40; p > 0.10), but general wind speed was negatively-correlated to PNC (r = -0.77; p = 0.018) and PD (r = -0.74; p = 0.021).

### Inflammatory response

#### Air quality detection and symptoms

The means for the specific detection and symptoms variables for LOW were significantly lower than HIGH for offensive odour detection [2.1 ± 0.5 vs. 2.8 ± 0.8: F(1,406) = 5.515; p = 0.019], dust and soot observation [1.7 ± 0.3 vs. 2.3 ± 0.5: F(1,140) = 4.340; p = 0.038], nasal and throat irritation [1.5 ± 0.3 vs. 1.9 ± 0.2: F(1,140) = 7.266; p = 0.007]. All other specific acute respiratory symptoms were not significantly different between HIGH and LOW (p > 0.10).

Offensive odour, and dust or soot, detection was significantly higher for both HIGH and LOW in-commute, compared to pre-commute and post-commute, [F(1,406) = 4.165 ; p < 0.031]. See Table 2. Nasal and throat irritation was significantly higher only for HIGH immediately-post-commute, compared to HIGH pre-commute and three-hour-post-commute, [F(1,140) = 7.545; p < 0.006]. See Table 2.

**Table 2:** Symptom and peak flow variables for routes of high (HIGH) and low (LOW) proximity to motorised traffic; direction and time

**Condition**	**HIGH**						**LOW**					
	**Inbound**			**Outbound**			**Inbound**			**Outbound**		
	**Pre**	**In**	**Post**	**Pre**	**In**	**Post**	**Pre**	**In**	**Post**	**Pre**	**In**	**Post**
Offensive Odour	1.09 ± 0.12	2.71^*,##^ ± 0.73	1.21 ± 0.15	1.12 ± 0.13	2.88^*,##^ ± 0.83	1.06 ± 0.11	1.18 ± 0.13	2.06^##^ ± 0.42	1.09 ± 0.15	1.18 ± 0.14	2.18^##^ ± 0.48	1.09 ± 0.11
Dust, Soot	1.06 ± 0.11	2.35^*,##^ ± 0.55	1.06 ± 0.11	1.06 ± 0.11	2.21^*,##^ ± 0.49	1.03 ± 0.12	1.18 ± 0.14	1.65^#^ ± 0.27	1.06 ± 0.11	1.15 ± 0.13	1.65^*,##^ ± 0.27	1.09 ± 0.21
Eye Irritation	1.06 ± 0.11	1.56 ± 0.24	1.06 ± 0.11	1.18 ± 0.14	1.65 ± 0.27	1.06 ± 0.10	1.18 ± 0.10	1.26 ± 0.16	1.09 ± 0.12	1.18 ± 0.14	1.35 ± 0.18	1.06 ± 0.12
Nose Irritation	1.38 ± 0.19	1.82^**^ ± 0.33	1.24 ± 0.15	1.24 ± 0.16	1.74 ± 0.30	1.12 ± 0.13	1.24 ± 0.15	1.53 ± 0.23	1.12 ± 0.13	1.09 ± 0.12	1.38 ± 0.19	1.12 ± 0.13
Throat Irritation	1.56 ± 0.24	2.00^**^ ± 0.40	1.41 ± 0.19	1.35 ± 0.18	2.09 ± 0.44	1.26 ± 0.16	1.38 ± 0.19	1.56 ± 0.24	1.26 ± 0.16	1.24 ± 0.15	1.56 ± 0.25	1.35 ± 0.18
Cough	1.32 ± 0.17	1.62 ± 0.26	1.32 ± 0.17	1.18 ± 0.14	1.71 ± 0.29	1.41 ± 0.20	1.29 ± 0.17	1.26 ± 0.16	1.26 ± 0.20	1.24 ± 0.16	1.35 ± 0.19	1.24 ± 0.15
Phlegm	1.26 ± 0.16	1.97 ± 0.38	1.26 ± 0.16	1.18 ± 0.15	1.94 ± 0.38	1.35 ± 0.18	1.47 ± 0.22	1.74 ± 0.30	1.21 ± 0.15	1.29 ± 0.17	1.76 ± 0.31	1.24 ± 0.13
Chest Tightness	1.12 ± 0.13	1.35 ± 0.18	1.09 ± 0.12	1.09 ± 0.13	1.47 ± 0.22	1.12 ± 0.13	1.09 ± 0.12	1.12 ± 0.13	1.12 ± 0.11	1.12 ± 0.13	1.15 ± 0.13	1.12 ± 0.13
Chest Wheeze	1.03 ± 0.11	1.24 ± 0.15	1.09 ± 0.12	1.06 ± 0.11	1.26 ± 0.16	1.15 ± 0.13	1.09 ± 0.13	1.09 ± 0.12	1.09 ± 0.13	1.06 ± 0.11	1.15 ± 0.11	1.09 ± 0.19
Headache	1.09 ± 0.12	1.21 ± 0.16	1.12 ± 0.13	1.12 ± 0.14	1.29 ± 0.17	1.09 ± 0.12	1.18 ± 0.14	1.09 ± 0.13	1.12 ± 0.16	1.18 ± 0.14	1.18 ± 0.14	1.03 ± 0.11
PFR (%Δ)	0.00 ± 0.00	1.28 ± 0.16	1.18 ± 0.14	0.00 ± 0.00	1.65 ± 0.27	1.63 ± 0.27	0.00 ± 0.00	1.76 ± 0.31	1.41 ± 0.20	0.00 ± 0.00	2.38 ± 0.57	2.14 ± 0.46

Values are mean ± standard deviation. Significance [from linear mixed models]: ^*^p < 0.05, ^**^p < 0.01 compared to LOW; ^#^p < 0.05, ^##^p < 0.01 compared to Pre. Direction is the return component of bicycle commute trip performance: Inbound = morning, ingress of CBD; Outbound = afternoon, egress of CBD. Time is the period relative to bicycle commute trip performance: Pre = immediately pre-commute; In = immediately-post-commute; Post = three hours post-commute. PFR = Peak flow rate. Values are given as mean, on a scale of incidence from 1 (very low) to 5 (very high). PFR is expressed as the percentage change from Pre values.

The means for detection and symptoms in-commute, compared to pre-commute and post-commute, were significantly higher for offensive odour detection (p ≤ 0.001), dust or soot detection (p ≤ 0.001), eye irritation (p ≤ 0.001), nasal irritation (p ≤ 0.001); throat irritation (p ≤ 0.001); phlegm production (p ≤ 0.001); and, chest tightness (p = 0.003). Cough and chest wheeze were significantly higher for in-commute p = 0.012, 0.017, respectively), but not the post-commute time period (p = 0.070, 0.176, respectively), compared to the pre-commute time period.

Participant age was positively-correlated with immediately-post-commute throat irritation (r = 0.78; p = 0.049) and phlegm production (r = 0.83; p = 0.024). Female participants, compared to males, reported significantly higher immediately-post-commute throat irritation (1.57 ± 0.88 versus 1.33 ± 0.68; p ≤ 0.001) and headache (1.14 ± 0.49 versus 1.06 ± 0.35; p = 0.005). No other participant characteristics were associated with these symptoms.

#### Peak flow rate

Peak flow rate was not significantly different within HIGH or LOW from pre-commute to immediately or three hours post-commute (p > 0.10) or, between HIGH and LOW post-commute. Female, compared to male, participants had a significantly lower mean peak flow rate across all conditions (447 ± 66 versus 584 ± 89 L·min; p ≤ 0.001). The mean intra-individual difference (and standard deviation) between PFR performance was 20.3 ± 11.3 L·min. See Table 2.

#### Sputum cell counts

Total and differential cell counts of valid sample sets [when compared to other values of healthy adults (35), 22 of 35; 63%] were not significantly different between LOW and HIGH, or between pre-commute and three hours post-commute. Specifically, there was no significant difference in neutrophil counts in HIGH three hours post-commute compared to in HIGH pre-commute, or in LOW three hours post-commute (p > 0.10). See Table 3. There was no correlation between neutrophil count and in-commute PNC or PD (r < 0.40; p > 0.10).

**Table 3:** Total and differential cell counts for routes of high (HIGH) and low (LOW) proximity to motorised traffic; direction and time

**Condition**	**HIGH**				**LOW**			
	**Inbound**		**Outbound**		**Inbound**		**Outbound**	
	**Pre**	**Post**	**Pre**	**Post**	**Pre**	**Post**	**Pre**	**Post**
Leukocyte (x e^6^ cells·mL^-1^)	1.36 ± 0.42	1.38 ± 0.43	1.23 ± 0.38	1.28 ± 0.39	1.40 ± 0.43	1.37 ± 0.42	1.44 ± 0.45	1.44 ± 0.45
Epithelial (x e^6^ cells·mL^-1^)	1.16 ± 0.30	1.19 ± 0.31	1.05 ± 0.27	1.10 ± 0.28	1.20 ± 0.31	1.17 ± 0.30	1.23 ± 0.32	1.23 ± 0.30
Columnar (x e^6^ cells·mL^-1^)	0.58 ± 0.18	0.59 ± 0.18	0.53 ± 0.16	0.55 ± 0.17	0.60 ± 0.19	0.59 ± 0.15	0.62 ± 0.22	0.62 ± 0.21
Squamous (x e^6^ cells·mL^-1^)	0.78 ± 0.30	0.79 ± 0.31	0.70 ± 0.27	0.73 ± 0.28	0.80 ± 0.31	0.78 ± 0.20	0.82 ± 0.29	0.82 ± 0.30
Macrophage (%)	59 ± 18	58 ± 18	59 ± 18	59 ± 18	59 ± 18	59 ± 20	58 ± 17	59 ± 18
Lymphocyte (%)	1.4 ± 0.4	1.5 ± 0.5	0.8 ± 0.3	0.9 ± 0.3	0.8 ± 0.2	1.3 ± 0.5	1.0 ± 0.3	1.0 ± 0.4
Neutrophil (%)	39 ± 12	40 ± 12	40 ± 12	40 ± 12	40 ± 12	39 ± 10	41 ± 11	40 ± 11
Eosinophil (%)	0.6 ± 0.2	0.5 ± 0.1	0.2 ± 0.1	0.1 ± 0.1	0.2 ± 0.1	0.7 ± 0.2	0.1 ± 0.1	0.1 ± 0.1

Values are mean ± standard deviation. Significance [from linear mixed models]: ^*^p < 0.05, ^**^p < 0.01 compared to LOW, ^#^p < 0.05, ^##^p < 0.01 compared to Pre. Total cell count values are presented as number of cells per mL of spontaneous sputum sample. Differential cell count values are presented as percentage of the total leukocyte cell count. Direction is the return component of bicycle commute trip performance: Inbound = morning, ingress of CBD; Outbound = afternoon, egress of CBD. Time point is relative to bicycle commute trip performance: Pre = immediately pre-commute; Post = three hours post-commute.

### Route preference and importance of components

Two-thirds of participants (23 of 35; 66%) preferred LOW compared to HIGH. For this preference, the components (in decreasing order by mean rating of importance) were safety (as a mean rating out of 5, and the standard deviation of the mean; 3.86 ± 0.19), health (3.06 ± 0.09), fitness (2.34 ± 0.11) and social (1.31 ± 0.20). Twelve participants (12 of 35; 34%) preferred HIGH compared to LOW, indicating the most important component as being time (3.63 ± 0.12).

## Discussion

The results of this project indicate that an informed route alteration designed to lower proximity to motorised traffic during bicycle commuting does significantly reduce exposure to combustion emissions (represented by PNC), as well as offensive odours, dust and soot. The route alteration does not affect factors of utility such as commute distance or duration. There was an increase in nasal and throat irritation, but no physiological inflammatory response for HIGH, compared to LOW, proximity to motorised traffic. It can be inferred that potential inhaled PNC is attributable predominantly to a difference in ambient PNC rather than a difference in heart rate or physical effort, and thus ventilation rate, or alternative routes.

### In-commute particle measurements

The exposure risk minimisation strategy of lowering proximity to motorised traffic while bicycle commuting has been shown to be effective under the circumstances of this study; mean and median PNC was significantly reduced with LOW compared to HIGH, which is in agreement with similar previous research [6,10,15]. The observation of the current study that the mean reduction in PNC was largest for the inbound, compared to outbound, commute of HIGH is also in agreement with previous urban measurement studies [37-39]. The reduction of median PNC level was generally smaller (although still significant) in magnitude compared to the reduction of mean PNC level, which suggests a strong influence by in-commute PNC peaks, which can be associated with road crossings and traffic control lights, on total commute PNC exposure. There was no significant difference between LOW inbound and outbound trips, indicating the influence by proximity to motorised traffic on PNC, particularly with the morning peak hour traffic, which is expected to be more time-concentrated than the afternoon peak [37-39].

Previously, in Brisbane, a mean PNC of 7.4 × e^3^ ppcc and a median PD of 40 nm (values strongly-associated with motor vehicle emissions) have been shown as background measurements [40]. More recently, PNC in Brisbane has been shown to have increased to a mean of 10.0 × e^3^ ppcc, and PD decreased to a median of 38 nm, however these levels are relatively-low compared with other cities worldwide [41] and generally do not reflect in-commute exposure but background concentrations [42,43]. A meta-analysis performed using 71 UFP studies of different environments showed typical mean PNC of 7.3 × e^3^ ppcc for urban background and 42.1 × e^3^ ppcc for roadside measurements, and indicated that higher proximity to motorised traffic is positively-associated with PNC [44]. In the current study, the median PD was not significantly different between HIGH and LOW, nor between inbound and outbound trips within either HIGH or LOW. That is, a higher mean PNC was not significantly associated with a lower mean PD, and vice versa, suggesting the in-commute measurement of fresh petrol emissions or nucleation events [41].

A recent meta-analysis of UFP exposure in-transit across different modes of transport indicates that cyclists are generally exposed to the lowest PNC of any mode [45]. Studies specifically comparing bicycle commute routes with high and low proximity to motorised traffic have indicated a mean PNC of 3.5 × e^4^ and 2.6 × e^4^ ppcc, respectively [45]. In comparison, motor vehicle passengers can be exposed to PNC 1.3 times higher than cyclists [46,47].

### Heart rate and physical effort

As bicycle commuting requires physical exercise, pulmonary ventilation rates of participants can be an important factor when determining the inhaled dose of UFP and therefore a toxic biological interaction. Ventilation rates of cyclists in previous studies have been approximately 2 to 4 times higher than motor vehicle passengers, though this rate is believed to be conservative [47,48]. An experiment study showed that particle deposition can be 4.5 times higher during moderate bicycling exercise compared to rest, in healthy individuals [49]. Inhaled mean particle count can be halved by using a pre-determined route alteration of minimal, compared to maximal, proximity to motorised traffic [33], similar to the current study results for mean PNC.

The correlation between heart rate and pulmonary ventilation rate during exercise is high and, while it varies between individuals, a predictable association can be made for an individual once a heart-rate ventilation association equation has been produced [50,51]. The current study did not include exercise testing to provide values for such an equation, however, the intention was to make intra-individual comparison of heart rates between route alterations to estimate if inhaled particle count was determined by PNC rather than pulmonary ventilation level. As heart rates were not significantly different between HIGH and LOW, it could be inferred that any potential difference in inhaled particle count would be attributable to differences in PNC rather than physical effort, and therefore ventilation rate, required for HIGH or LOW. A previous study in the same geographical region by the current research group showed that estimated ventilation rates (via heart rate-ventilation association curves produced with exercise testing) were not significantly different between popular bicycle commute routes of low and high proximity to motorised traffic [33].

The mean distance and duration of commutes were not significantly increased from the alteration to LOW from HIGH, therefore not increasing overall exposure to motorised traffic emissions due to an increased exposure period. As commute distance or duration is not increased, the utility of an altered bicycle commute route to lower proximity to motorised traffic emissions has been demonstrated, in this project, as practical for individuals rating time as an important feature of a commute route.

### Climate variation

In the current project, the measured meteorological variables were not significantly different between HIGH and LOW. The diurnal variation of climate potentially produced a higher mean temperature and lower mean humidity for the outbound (afternoon), compared to the inbound (morning), commute. The influence that these differences in temperature and humidity have when comparing air quality measures of HIGH and LOW is negligible as inbound and outbound trips were performed in equal measure for HIGH and LOW. The significantly-higher PNC in HIGH inbound compared to HIGH outbound could be attributable to nucleation inhibition occuring in the HIGH outbound (afternoon) with the circumstances of higher temperature and lower humidity [52]. Previous study conditions of lower temperature and higher humidity have been shown to produce secondary particle production and an increase in PNC [53-56], such as the circumstances in the HIGH inbound (morning) commute in the current project.

### Air quality detection

The association of air pollution exposure perception and symptoms during active transport has had limited attention in previous research. In the current project, the detection of offensive odours and particles, dust and soot was higher in-commute for HIGH compared to LOW, which was generally similar to actual air quality (indicated by the mean PNC). Previously, odorous traffic fumes and visible dust or soot annoyed community members despite pollutant levels being compliant with current World Health Organisation (WHO) recommendations, and irritation of the eyes was significantly associated with respondent rating of air quality [57]. The adequate perception of in-commute air quality, via the detection of offensive odours and particles, dust or soot, could assist individuals to re-consider their commute route to decrease exposure (as this study has found to be effective) when and where appropriate.

### Physiological inflammatory response

In the current project, in conjunction with the significantly higher PNC for HIGH compared to LOW, there was an increase in offensive odour detection and nasal irritation, but no acute respiratory symptoms were increased immediately-post-commute and three-hours-post-commute. While personal NO_X_ exposure was not monitored, it is probable that NO_X_ concentrations were substantially higher with HIGH compared to LOW due to the strong association of NO_X_ to motorised traffic emissions and particle number [58]. However, despite an increased offensive odour detection with HIGH compared to LOW, acute respiratory symptoms that may be associated with elevated NO_X_ exposure concentrations (including nasopharyngeal irritation, dyspnoea and tussis; observed previously with research of indoor air quality [59]) were not increased post-commute or decreased for LOW compared to HIGH.

In the current project there was no significant change in peak expiratory flow rate or neutrophil counts, either pre-commute to post-commute or in LOW compared to HIGH. Previously, healthy and asthmatic adults exposed to a mean PNC of 1.45 × e^5^ ppcc during 2 hours of intermittent exercise did not exhibit significant differences in sputum neutrophil counts immediately or four hours post-exposure [27]. Similarly, healthy and asthmatic adults exposed to a mean PNC of 4.77 × e^6^ ppcc during rest and exercise did not have significant differences in respiratory symptoms or sputum neutrophil counts, however did have a reduction in maximal mid-expiratory flow rate twenty-one hours post-exposure [4].

While the results of this project did not indicate any acute health effects from the variables measured, the PNC exposure levels (HIGH = 3.3 × e^4^ ppcc, ~40 mins and LOW = 1.8 × e^4^ ppcc, ~40 mins) surpass previous levels observed to increase systemic markers of inflammation in healthy individuals exercising intermittently for a longer duration (1.1 × e^4^ ppcc, 120 mins) [60]. Exposure at higher levels of UFP and for longer durations than the current project has elicited increases in lower airway inflammatory mediators [61] and systemic markers of inflammation [28], oxidative DNA damage [43], and decreased lung function from airway inflammation [6]. Therefore, it is reasonable that the mean PNC exposure levels and durations for HIGH and LOW were too low to significantly affect the specific acute health variables measured in the current project.

Sputum neutrophils, obtained from the lower airways, have been used previously as a biomarker for airway inflammation, but these cells can have a low association with lung function and respiratory symptoms [20]. The utility of repeated sputum induction on cell counts over a 24-hour period has been questioned [62]. One project has shown no significant changes in sputum cell differential counts for healthy individuals in response to PNC exposure during rest and exercise (≤ 6.9 × e^6^ ppcc, 120 mins). However, in asthmatics following a similar protocol, PNC was associated with a significant increase in alveolar macrophages by 11% compared to filtered air [4]. A physiological inflammatory response was not indicated in the current project as there was no significant change in neutrophil count.

Significant effects of UFP exposure on symptoms, pulmonary function, and markers of airway or systemic inflammation are not yet confirmed [63]. The mechanisms of these effects for inhaled UFP are not yet known, but these particles have significantly higher pulmonary inflammatory effects compared to coarser particles at equal mass dose [7,45,64]. Regardless, the strategy of altering a bicycle commute route to lower proximity to motorised traffic continues to be shown as effective at substantially reducing exposure to vehicle-emitted air pollution such as UFP, even if evidence of a health-protective advantage in healthy individuals has currently not yet been determined [65].

### Participant preference of commute alteration

Previous research by the current research group showed that a large cohort (n = 155) of frequent inner-city bicycle commuters would consider re-routing their commute to lower proximity to motorised traffic, if this was proven to be an appropriate and effective risk management strategy and dependent on route factors of safety (i.e. greater riding space or visibility) as well as time (i.e. duration of commute) [22]. Off-road routes allow a reduction of the proximity to motorised traffic and thus improved physical safety (along with reduced air pollution exposure risk), but these routes are typically less direct and take a longer time to complete. However, this was not the case in this study, as commute distance and duration were not affected by the alteration of the commute route to lower proximity to motorised traffic. The participants of the current study typically used a bicycle commute route of high proximity to motorised traffic, and some preferred their typical route (deemed as HIGH) compared to LOW after participation in this study, due primarily to the component of time. Conversely, LOW was preferred over HIGH by most participants due to reasons of health (that is, to avoid air pollution) and safety of the participant. Therefore, the development of appropriate infrastructure and educational schemes would be desirable to implement a sustainable bicycle commuting environment, to assist an individual to manage their own air pollution exposure risk, as necessary.

### Limitations

At the time of conducting this project, the personal UFP monitor used was novel for field research and therefore precedent references were not available, however, previous similar field research [33], personal monitoring of children [66] and device inter-comparison laboratory testing [67] has now been published. In the current project, device correction factors for the monitors used were obtained in controlled conditions against a standard device (see Methods), and the data observed was accepted to be valid. The design of a unique questionnaire (such as that used for symptom reporting in the current project) without a precedent model available for reference will include a factor of unknown validity and reliability. As the questionnaire was self-administered, respondent misunderstandings could have occurred due to question misinterpretation. Questionnaire response measurement error could have resulted as participants could not be blinded to the routes of HIGH or LOW expected decreased. The performance of peak flow measuring and spontaneous sputum sampling were dependent on participant competence and therefore could include measurement error. Verbal and written instructions were provided to participants at induction (see Additional file 1), but field performance was not supervised and therefore cannot be validated; however, specific post-completion quality controls were used (see relevant sub-sections in Methods).

## Conclusions

Exposure to ultrafine particles, typically associated with combustion emissions of motorised traffic, can be significantly reduced by lowering proximity to motorised traffic without significantly increasing commute distance or duration whilst bicycle commuting. Regulatory authorities encouraging bicycle commuting participation should educate participants in air quality and risk management, and also ensure practical consideration when creating bicycle commuting routes, to maximise participation rates yet minimise any health risk consequent to chronic, frequent in-commute exposure to motorised traffic emissions.

## Abbreviations

CBD: Central business district; CPC: Condensation particle counter; PEF: Peak expiratory flow; PD: Particle diameter; PM: Particulate matter; PNC: Particle number concentration; ppcc: Particles per cubic centimetre; UFP: Ultrafine particle.

## Competing interests

The authors declare that they have no competing interests.

## Authors’ contributions

TCH conceived and designed the study, recruited participants, collected and analysed all data and drafted the manuscript; LM supervised study design, exposure data collection and analysis, and manuscript revision; IS supervised study design, clinical data analysis, and manuscript revision; MH assisted with study design, and clinical data collection and procession; RJ supervised study design, exposure data collection, and data analysis and manuscript production and revision; CS assisted and/or supervised study design, clinical data collection and procession, exposure data collection, data analysis and manuscript revision. All authors read and approved the final manuscript.

## Supplementary Material

Additional file 1 Participant Instructions. Click here for file

Additional file 2 Participant checklist & data sheet (per day / return trip). Click here for file
