# Supplementary material for: Utility of an alternative bicycle commute route of lower proximity to motorised traffic in decreasing exposure to ultra-fine particles, respiratory symptoms and airway inflammation – a structured exposure experiment
Source: Environ Health. 2013 Apr 8;12:29. doi: 10.1186/1476-069X-12-29 (PMC4177132; doi:10.1186/1476-069X-12-29)
Supplement: Additional file 1 — Participant Instructions. [file 1476-069X-12-29-S1.docx]

**PARTICIPANT INSTRUCTIONS**

*Please try to avoid any potential sources of air pollution or allergens, within reason, such as exhaust, smoke, harsh chemicals, etc.*

**TESTS**

**Questionnaire (1^st^)**

*Complete Questionnaire to the best of your ability.*

**Peak Flow Test (2^nd^)**

1. *Firmly insert white cardboard mouthpiece into meter.*
2. *Move red pointer to ‘60L/MIN’ end of slot (closest to mouth)*
3. *Hold meter lightly without obstructing red pointer movement*
4. *With a straight back: take deepest possible breath, seal lips around mouthpiece, blow into mouthpiece as hard and fast as you can (without coughing or spitting)*
5. *Take meter from your mouth and note number opposite red pointer on sheet provided*
6. *Repeat steps 2 to 5 two more times.*

**Sputum Sample (3^rd^)**

1. *Rid mouth of saliva,*
2. *Cough vigorously three times,*
3. *“Huff”(exhale forcefully),*
4. *“Hock”(clear back of throat),*
5. *Carefully spit into tube (without splashing)*
6. *Repeat steps 1 to 5 two more times*
7. *Shake tube to mix sputum and preservative (within tube).*

**EQUIPMENT**

**GPS (‘**QSTARZ Travel Recorder X’**)**

1. Switch on side from ‘OFF’ to ‘LOG’, immediately before commute.
2. Position GPS close to outside of bag for clear satellite signal.
3. Switch off side from ‘LOG’ to ‘OFF’, three hours after commute.

**Air Pollution Monitor (**‘Philips NanoTracer’**)**

1. Switch on side from ‘OFF’ to ‘ADV’, *immediately before commute (with 2 minutes to warm-up)*.
2. Position rubber extension tube inlet close to mouth/nose (e.g. collar).
3. Switch off side from ‘ADV’ to ‘OFF’, *three hours after commute*.

**Heart Rate Monitor (**‘POLAR’**)**

1. Place band around chest, adding a little water (or sweat) to electrodes if possible to improve signal.
2. Place watch around wrist, hold down big red button on watch and wait for heart rate to appear and update, *immediately before commute*.
3. Press stop button (‘’), *three hours after commute*.

***Please try to leave the NanoTracer, Polar HR, and GPS devices close to your person and operating for the three hours post-commute, but away from heat, moisture and sunlight.*** ***If you have any problems, please call Tom on* 0402 283 275 *anytime****.*
