# Supplementary material for: Utility of an alternative bicycle commute route of lower proximity to motorised traffic in decreasing exposure to ultra-fine particles, respiratory symptoms and airway inflammation – a structured exposure experiment
Source: Environ Health. 2013 Apr 8;12:29. doi: 10.1186/1476-069X-12-29 (PMC4177132; doi:10.1186/1476-069X-12-29)
Supplement: Additional file 2 — Participant checklist & data sheet (per day / return trip). [file 1476-069X-12-29-S2.doc]

**PARTICIPANT CHECKLIST & DATA SHEET (per day / return trip)**

**** (*Please enter Time of commute finish, and tick-off tests when completed*) ****

| **Event** | **Time** | **Symptom Reporting Questionnaire** | **Peak Flow Test**  **(Lung Function)**  via **‘Blast**’ | **Sputum Sample** via ‘**Cough,Huff,Hock’** |
| --- | --- | --- | --- | --- |
| **1^st^ Trip –**  **Pre-Commute** | [ __ __ : __ __ ] | Q1. |  |  |
| **1^st^ Trip –**  **Post-Commute** | [ __ __ : __ __ ] | Q2. |  |  |
| **1^st^ Trip –**  **3 hrs Post-Commute** | [ __ __ : __ __ ] | Q3. |  | **DO NOT DO** |
| **2^nd^ Trip –**  **Pre-Commute** | [ __ __ : __ __ ] | Q1. |  |  |
| **2^nd^ Trip –**  **Post-Commute** | [ __ __ : __ __ ] | Q2. |  |  |
| **2^nd^ Trip –**  **3 hrs Post-Commute** | [ __ __ : __ __ ] | Q3. |  | **DO NOT DO** |

DATE: ‘HIGH’/’LOW’ Traffic Route:

| **Symptom Reporting Questionnaire** |
| --- |

**PLEASE ANSWER THE QUESTIONS USING THE FOLLOWING SCALE (‘O’ for 1^st^ Trip, ‘X’ for 2^nd^ Trip of same route):**

**1 = Never 2 = Rarely 3 = Sometimes 4 = Often 5 = Always**

| **Q1. Within the hour before your cycle commute...** | | **NEVER** | |  | | | **ALWAYS** | |
| --- | --- | --- | --- | --- | --- | --- | --- | --- |
|  | Did you notice the presence of offensive odours? -------------------------------------- | **1** | **2** | | **3** | **4** | | **5** |
|  | Did you experience irritation of the eyes? ------------------------------------------------ | **1** | **2** | | **3** | **4** | | **5** |
|  | Did you experience irritation of the nose? ------------------------------------------------ | **1** | **2** | | **3** | **4** | | **5** |
|  | Did you experience irritation of the throat? ---------------------------------------------- | **1** | **2** | | **3** | **4** | | **5** |
|  | Did you experience an increased prevalence or incidence of cough? -------------- | **1** | **2** | | **3** | **4** | | **5** |
|  | Did you experience an increased prevalence  or incidence of phlegm production? --------------------------------------------- | **1** | **2** | | **3** | **4** | | **5** |
|  | Did you experience tightening in the chest? ---------------------------------------------- | **1** | **2** | | **3** | **4** | | **5** |
|  | Did you experience wheezing in the chest? ----------------------------------------------- | **1** | **2** | | **3** | **4** | | **5** |
|  | Did you have a headache? -------------------------------------------------------------------- | **1** | **2** | | **3** | **4** | | **5** |
|  | Did you notice any clouds of particles, dust or soot? ----------------------------------- | **1** | **2** | | **3** | **4** | | **5** |

**Please turn over… →**

| **Q2. During your cycle commute...** | | **NEVER** | |  | | | **ALWAYS** | |
| --- | --- | --- | --- | --- | --- | --- | --- | --- |
|  | Did you notice the presence of offensive odours? -------------------------------------- | **1** | **2** | | **3** | **4** | | **5** |
|  | Did you experience irritation of the eyes? ------------------------------------------------ | **1** | **2** | | **3** | **4** | | **5** |
|  | Did you experience irritation of the nose? ------------------------------------------------ | **1** | **2** | | **3** | **4** | | **5** |
|  | Did you experience irritation of the throat? ---------------------------------------------- | **1** | **2** | | **3** | **4** | | **5** |
|  | Did you experience an increased prevalence or incidence of cough? -------------- | **1** | **2** | | **3** | **4** | | **5** |
|  | Did you experience an increased prevalence  or incidence of phlegm production? --------------------------------------------- | **1** | **2** | | **3** | **4** | | **5** |
|  | Did you experience tightening in the chest? ---------------------------------------------- | **1** | **2** | | **3** | **4** | | **5** |
|  | Did you experience wheezing in the chest? ----------------------------------------------- | **1** | **2** | | **3** | **4** | | **5** |
|  | Did you have a headache? -------------------------------------------------------------------- | **1** | **2** | | **3** | **4** | | **5** |
|  | Did you notice any clouds of particles, dust or soot? ----------------------------------- | **1** | **2** | | **3** | **4** | | **5** |

| **Q3. Within the three hours after your cycle commute...** | | **NEVER** | |  | | | **ALWAYS** | |
| --- | --- | --- | --- | --- | --- | --- | --- | --- |
|  | Did you notice the presence of offensive odours? -------------------------------------- | **1** | **2** | | **3** | **4** | | **5** |
|  | Did you experience irritation of the eyes? ------------------------------------------------ | **1** | **2** | | **3** | **4** | | **5** |
|  | Did you experience irritation of the nose? ------------------------------------------------ | **1** | **2** | | **3** | **4** | | **5** |
|  | Did you experience irritation of the throat? ---------------------------------------------- | **1** | **2** | | **3** | **4** | | **5** |
|  | Did you experience an increased prevalence or incidence of cough? -------------- | **1** | **2** | | **3** | **4** | | **5** |
|  | Did you experience an increased prevalence  or incidence of phlegm production? --------------------------------------------- | **1** | **2** | | **3** | **4** | | **5** |
|  | Did you experience tightening in the chest? ---------------------------------------------- | **1** | **2** | | **3** | **4** | | **5** |
|  | Did you experience wheezing in the chest? ----------------------------------------------- | **1** | **2** | | **3** | **4** | | **5** |
|  | Did you have a headache? -------------------------------------------------------------------- | **1** | **2** | | **3** | **4** | | **5** |
|  | Did you notice any clouds of particles, dust or soot? ----------------------------------- | **1** | **2** | | **3** | **4** | | **5** |

**Peak Flow Test: Results (of 3 Trials)**

| ***CBD One-Way Trip 1*** | | | | ***CBD One-Way Trip 2*** | | | |
| --- | --- | --- | --- | --- | --- | --- | --- |
| ***Trial:*** | *1* | *2* | *3* | ***Trial:*** | *1* | *2* | *3* |
| ***Pre-Cmt*** [ : ] |  |  |  | ***Pre-Cmt*** [ : ] |  |  |  |
| ***Post-Cmt*** [ : ] |  |  |  | ***Post-Cmt*** [ : ] |  |  |  |
| ***3 hrs Post*** [ : ] |  |  |  | ***3 hrs Post*** [ : ] |  |  |  |
